# Clinical characteristics of newly diagnosed tuberculosis patients with respiratory failure

**DOI:** 10.1186/2197-425X-3-S1-A391

**Published:** 2015-10-01

**Authors:** C Permpikul, S Tongyoo, W Lekpittaya, P Thamrongpairoj

**Affiliations:** Division of Critical Care, Medicine, Bangkok, Thailand

## Introduction

Respiratory failure from tuberculosis is not as common as other community acquired infection. Delay in diagnosis of tuberculosis thus poses significant impact on disease management and ICU infection control. We report here the clinical characteristics of this condition in order to render better diagnosis and early management.

## Methods

A retrospective study in inpatients whose a new diagnosis of tuberculosis was made after admission. The information reviewed included clinical characteristics, laboratory results and outcomes.

## Results

Among 738 patients with tuberculosis who were admitted during January 2011 and December 2013, 60 were newly diagnosed. Of these, 35 (58.3%) patients had respiratory failure. When comparing those who had respiratory failure (RF) and those who did not (no RF), there was no significant difference in age (RF 47.3 ± 16.9 VS no RF 44.5 ± 15.8, P = 0.51), male sex (71.4% VS 68.0%, P = 0.76), BMI (17.9 ± 4.VS 018.8 ± 4.2, P = 0.43) and underlying diseases. Less cough was noted in RF group (odd ratio 4.55, 95%CI 1.19-100.00, P = 0.04). RF patients had higher admission SOFA score (4.3 ± 3.6 VS 2.2 ± 2.1, P = 0.007) and the score progressively increased in 45.7% of patients. The presence of military infiltration or cavitation were found in only one third of the patients. With certain limitation, conventional anti-TB regimens were given in only 51.4% of RF patients. Consequently, Patients with RF had higher hospital mortality (51.4% VS 12%, P = 0.002).

## Conclusions

Substantial proportion of patients with newly diagnosed tuberculosis had respiratory failure. This condition was associated with higher hospital mortality. Notably, RF patients had less prominent presenting symptom, progressive deteriorated and inability to receive potent anti-TB medication.

